# The Western Veterinary College, Kansas City, Mo.

**Published:** 1901-04

**Authors:** 


### THE WESTERN VETERINARY COLLEGE, KANSAS CITY, MO.

The commencement exercises were held on Thursday, March 21st, at the Jackson County Medical Society Hall. After the invocation by the Rev. F. L. Bowen, the faculty address was made by Dr. St. Elmo Sanders. The valedictory was given by Joseph H. Hanna; the class prophecy by George W. Smith; the class of 1902 by Harvey E. McGee. The diplomas were awarded by Dr. J. H. Wattles, Sr. The following were awarded diplomas: John W. Chenoweth, Albany, Mo.; Arthur T. Coleman, Kansas City, Mo.; Arthur Hamilton Hart Lewis, Ashuelot, N. H.; Charles W. Hobbs, Kensington, Kan.; Robert N. McCarroll, Fort Collins, Col.; William E. Main, Canton, Ill.; Joseph H. Hanna, Burlington, Kansas; Theodore Lane, Iosco, Mich.; John H. Lomax, St. Joseph, Mo.; Sidney E. Bock, Denver, Col.; George W. Smith, Manchester, N. Y.; John F. Smith, Perham, Minn.; John W. Hefferman, Gregory, Mich.; William N. Waugh, Springfield, Mo.; William J. Hauser, Carthage, Mo.; Joseph D. Cluts, Civer, Ill.; Frank E. Judd, Zumbrota, Minn.; James McRobert, Council Bluffs, Iowa; Elijah G. Cluts, Civer, Ill.; Ralph C. Nickerson, Zumbrota, Minn.; William T. Chrisman, Cleburne, Texas; Herman H. Wolf, Canton, Ill.; William T. Duncan, Springfield, Mo.

